# Suppressor analysis in *Synechococcus elongatus* PCC7942 reveals key roles of (p)ppGpp in survival and nucleotide homeostasis

**DOI:** 10.3389/fmicb.2026.1860886

**Published:** 2026-06-03

**Authors:** Antonio Llop, Sirine Bibak, Raquel Cantos, Paloma Salinas, Lorena Tremiño, Asunción Contreras

**Affiliations:** Departamento de Fisiología, Genética y Microbiología, Universidad de Alicante, San Vicente del Raspeig, Spain

**Keywords:** cyanobacteria, GTP homeostasis, GuaB3, Rel, S2, *synpcc7942_0187*

## Abstract

Despite the environmental and biotechnological importance of cyanobacteria, most of the molecular mechanisms involved in maintaining cellular homeostasis and adaptation to environmental changes remain unknown or hardly studied in comparison to other bacterial groups. This is the case of signalling by (p)ppGpp (guanosine penta- and tetra-phosphate), a complex process involving the reorganization of gene expression, which is conserved in bacteria and plants. We show here that some levels of (p)ppGpp are required for *S. elongatus* viability under standard laboratory growth conditions and exploited this finding to identify suppressor mutations partially restoring viability. We found that, as in firmicutes, the purine biosynthesis pathway appears to be the main target of (p)ppGpp signalling. In addition, the translation machinery also emerged as a key cyanobacterial target, a result facilitated by the occurrence of a small inversion targeting residues 15His-16Phe at the essential ribosomal subunit S2. Finally, the identification of a putative nucleotide signaling protein, encoded by *synpcc7942_0187,* points to regulatory peculiarities involved in (p)ppGpp signalling in cyanobacteria. This work paves the way for a molecular understanding of the functions and targets of (p)ppGpp in cyanobacteria, anticipating novel regulatory mechanisms unique to this important and understudied bacterial group.

## 1 Introduction

The second messengers (p)ppGpp (guanosine penta- and tetra-phosphate) were discovered as magic spots induced by amino acid starvation in *E. coli* (Cashel and Gallant, 1969). Subsequent studies (Horvatek et al., 2020; Irving and Corrigan, 2018; Kessler et al., 2017; Romand et al., 2022; Ruwe et al., 2019; Urwin et al., 2024) reported the induction of the so-called stringent response by nutrient limitation and different types of stress in other organisms. This global stress response program involves the reorganization of gene expression and is broadly conserved in bacteria and plants (Atkinson et al., 2011; Chi and Zhou, 2023; Ito et al., 2017). In cyanobacteria and plants nitrogen deprivation also induces (p)ppGpp accumulation (Akinyanju and Smith, 1979; Field, 2018; Friga et al., 1981; Romand et al., 2022; Zhang et al., 2013), but so far the most efficient trigger of the stringent response appears to be inhibition of photosynthetic activity during night periods (Hood et al., 2016; Puszynska and O’Shea, 2017; Surányi et al., 1987).

Initial studies fuelled the view of the (p)ppGpp alarmones as a biphasic switch between relaxed and stringent conditions (Jones et al., 1992). However, the multiplicity and diversity of (p)ppGpp targets amongst different bacterial groups and the growing evidence that (p)ppGpp controls multiple processes under optimal or standard laboratory culture conditions (Bange et al., 2021; Hauryliuk et al., 2015), supports the importance of (p)ppGpp as secondary messengers continuously regulating cell physiology and homeostasis (Fernández-Coll and Cashel, 2020; Irving et al., 2021; Kriel et al., 2012; Potrykus et al., 2026; Potrykus et al., 2011; Puszynska and O’Shea, 2017; Zhang et al., 2013).

In *Synechococcus elongatus* PCC7942 (hereafter *S. elongatus*), a model cyanobacterium for genetic studies (Holtman et al., 2005; Hou et al., 2023; Labella et al., 2020b, 2020a; Perrin et al., 2025; Rubin et al., 2015; Tremiño et al., 2022) (p)ppGpp is required for normal cell physiology even during growth under continuous light (Hood et al., 2016; Puszynska and O’Shea, 2017; Rubin et al., 2015), standard laboratory conditions that are considered non-stressful. Furthermore, (p)ppGpp deficiency results in dramatic impacts on cell physiology and global gene regulation, leading to higher levels of total RNA and to increases on global transcription and translation rates (Puszynska and O’Shea, 2017). Overproduction of (p)ppGpp also has very dramatic consequences (Hidese et al., 2023; Llop et al., 2023).

Multiple proteins with high affinity for (p)ppGpp or direct targets are known in different bacteria, many of them GTPases or GTP binding proteins (Bennison et al., 2021; Hauryliuk et al., 2015; Kriel et al., 2012; Osaka et al., 2020; Pausch et al., 2018). In addition, indirect regulation by (p)ppGpp involves global reprogramming of gene expression, achieved by distinct molecular strategies in proteobacteria and firmicutes, where they have received most of the attention (Hauryliuk et al., 2015; Liu et al., 2015; Potrykus et al., 2011). In the former case, the main function of (p)ppGpp appears to be decreasing and reprogramming transcription while in *B. subtilis* and other gram-positive bacteria it would be maintenance of GTP homeostasis.

In *E. coli* (p)ppGpp binds directly to RNA polymerase (Ross et al., 2016, 2013; Sanchez-Vazquez et al., 2019; Weaver et al., 2023; Zuo et al., 2013) and RNApol bound to (p)ppGpp works with the transcriptional regulator DksA to control a rather large regulon (Gourse et al., 2018; Parshin et al., 2015; Paul et al., 2004; Ross et al., 2016). In *B. subtilis* the transcriptional regulator CodY directly senses GTP to control nucleotide related operons (Shivers and Sonenshein, 2004). Neither DksA nor CodY, sensing guanosine nucleotides in proteobacteria and firmicutes, respectively (Anderson et al., 2023; Levdikov et al., 2017) have homologs in cyanobacteria, anticipating a yet unknown mechanism for transcriptional reprogramming in this bacterial group, where the mechanisms by which (p)ppGpp function to maintain cell homeostasis have not been addressed.

Genetic screens for mutations suppressing (p)ppGpp deficiency led to the identification of critical targets in both proteobacteria and firmicutes. Pioneer searches for suppressors of (p)ppGpp deficiency identified in *E. coli* the *rpoB* and *rpoC* genes (Hernandez and Cashel, 1995), encoding the β and β’ subunits of RNA polymerase, and in *B. subtilis* the *guaB*, *guaA* and *gmk* genes, encoding enzymes involved in GTP biosynthesis as well as the GTP-binding regulator *codY* (Kriel et al., 2012). However, the use of a second strain of *B. subtilis* with different nutritional requirements allowed the identification of (p)ppGpp^0^ suppressor mutations at *rpoB* and *rpoC* genes (Osaka et al., 2020), calling attention to the importance of the experimental context to gain insights into the main biological roles of (p)ppGpp in bacteria (Dworkin, 2023; Hauryliuk et al., 2015; Irving et al., 2021).

In cyanobacteria (p)ppGpp levels depend on just one enzyme, the bifunctional (p)ppGpp synthetase/hydrolase Rel and thus the obtention of (p)ppGpp^0^ mutants would require inactivation of a single gene. However, we could not obtain completely segregated mutants carrying the inactive *rel* allele in *S. elongatus*, making us wonder on the possible reasons, the essentiality of Rel and the role of (p)ppGpp under standard laboratory conditions.

We have shown here that some levels of (p)ppGpp are required for *S. elongatus* viability and subsequently exploited this finding to identify suppressor mutations partially restoring viability. The purine biosynthesis pathway and the translation machinery emerged as the main (p)ppGpp regulatory targets in cyanobacteria. This work paves the way for a molecular understanding of the functions and targets of (p)ppGpp in cyanobacteria, anticipating novel regulatory mechanisms unique to this important and under studied bacterial group.

## 2 Materials and methods

### 2.1 Cyanobacteria culture conditions and strain generation

Cultures were routinely grown in blue–green algae BG11 medium BG11_0_ supplemented with 17.5 mM sodium nitrate (NaNO₃) and 10 mM HEPES/NaOH (pH 7.8) (Rippka et al., 1979) at 30 °C under constant cool white fluorescent light, either in liquid cultures (150 rpm, 70 μmol photons m^−2^ s^−1^) or on plates (50 μmol photons m^−2^ s^−1^). Solid media contained 1.5% (w/v) agar and 0.5 mM sodium thiosulfate (Na₂S₂O₃). When appropriate, chloramphenicol (Cm, 3.5 μg/mL) or ampicillin (Ap, 10 μg/mL) were added.

Liquid cultures were grown in baffled flasks containing 30 mL of medium. Optical density at 750 nm (OD_750_) was measured as needed in 1 mL samples using an Ultrospec 2,100 Pro UV–Vis Spectrophotometer (Amersham Biosciences, Amersham, UK).

To test growth on solid media, exponentially growing cultures were adjusted to 0.5 (OD_750_) before dropping 5 μL of the cell suspensions or serial dilutions (5^−1^, 10^−1^, and 10^−2^) onto BG11 plates. Plates were incubated under standard conditions for 3 days and then photographed using a Nikon camera at the default parameters.

Transformations were performed essentially as described in Taton et al. (2020). Strains were transformed with plasmid pUC-ΔSynpcc7942_1377(rel)-Cm^R^ (Hood et al., 2016) and verified by PCR analysis. The primer pairs used (Table S1) were relA-2F/relA-2R for verification of Δ*rel:cat* allele replacement and rel-3F/relA-2R for verification of complete removal of *rel* allele.

To obtain strains with spontaneous suppressor mutations, transformant colonies were streaked onto the same selective Cm-containing plates, checked for Ap-sensitivity, and the double recombinant clones subjected to successive passages on selective medium (Cm) at 30 or 40 °C. Fully segregated clones were identified by PCR at different stages of the screening process.

### 2.2 Genomic DNA extraction and purification

Cultures were grown until the end of exponential growth phase (OD_750_ of 0.8), pelleted at 12000 × g for 10 min and resuspended in 400 μL of TE buffer (10 mM Tris-HC1; 1 mM EDTA, pH 8). 200 μL of muffled glass beads (100 μm), 20 μL of 10% SDS, and 450 μL of phenol:chloroform (1:1) were added to disrupt cells using three cycles of 60/60 s at a speed of 5 m/s and at 4 °C in a high-speed homogenizer Minibeadbeater. After centrifugation (15,000 g for 15 min at 4 °C) the supernatant fraction was transferred to a fresh tube and one volume of phenol:chloroform:isoamyl alcohol (25:24:1) was added. tubes were vigorously vortexed, centrifuged (15,000 g for 10 min at 4 °C) and the aqueous phase transferred to a fresh 1.5 mL tube before addition of one volume of chloroform:isoamyl alcohol (24:1), followed by vortexing and centrifugation (15,000 g for 10 min at 4 °C). The aqueous phase was then transferred to a fresh 1.5 mL tube and DNA was precipitated by adding 0.1 volume of NaOAc 3 M (pH 5.2) and 2 volumes of cold absolute ethanol, mixing with vortex and incubating O/N at −20 °C. DNA was then pelleted by centrifugation at 15000 g for 15 min at 4 °C, washed twice with ethanol 70%, dried in a speed-vac (10–20 min) and resuspended in 50 μL of H_2_O mQ. 1 μL of RNAase (10 mg/mL) was used per 20 μL of DNA at room temperature (20 min) before DNA purification using the NZYGelpure kit from NZYTech. DNA was quantified with qubit (ThermoFisher scientific) and pooled in equal amounts in groups of two to four samples.

### 2.3 Next-generation (NGS) and sanger sequencing

Genomic DNA sequencing was performed by Macrogen Inc. (Seoul, South Korea). Libraries were prepared using the Nextera DNA XT kit and sequenced on an Illumina NovaSeq 6,000 platform to generate 150 bp paired-end reads (~3 Gb per sample). Point mutations were identified through read mapping, variant calling, and annotation against the *S. elongatus* NCBI reference genome (accession number JACJTX01). Sequences were subsequently compared with those of the parental strains to identify unique mutations. Allelic status was confirmed by Sanger sequencing using the primer pairs (Table S1) rpsB-1F/rpsb-1R for *rpsB*, guaA-1F/guaA-1R for *guaA*, guaB-1F/guaB-1R for *guaB3*, and corC-1F/corC-1R for *_0187*.

### 2.4 Confocal microscopy and flow cytometry

Micrographs were taken using a Zeiss LSM800 confocal laser scanning microscope. 5 μL of samples were placed on 2% low-melting-point agarose pads. Auto-fluorescence (coloured as red) was measured using the microscopy settings ex 640 nm/em 650 + nm. To determine the percentage of cells containing septa, images were manually analyzed using ImageJ v1.54g.

Flow cytometry data was acquired in a Spectral Flow Cytometry (Cytek Aurora) and analysed using the web-based tool Floreada.^1^ To determine living cells size, events were filtered by the signal obtained from the 640 nm laser (minimum value > 10^4^).

### 2.5 Protein domain identification

The highest-scoring motifs of GuaA from *S. elongatus* (*synpcc7942_0189*) or *B. subtilis* (*BSU06360*), and ORF0187 from *S. elongatus* (*synpcc7942_0187*) were identified using the SSDB Motif Search tool available in KEGG database. For GuaB/GuaB3 proteins, motif identification was based on the analysis reported by Hernández-Gómez et al. (2023).

## 3 Results

### 3.1 Inactivation of the *rel* gene in *S. elongatus*

**Figure 1:**
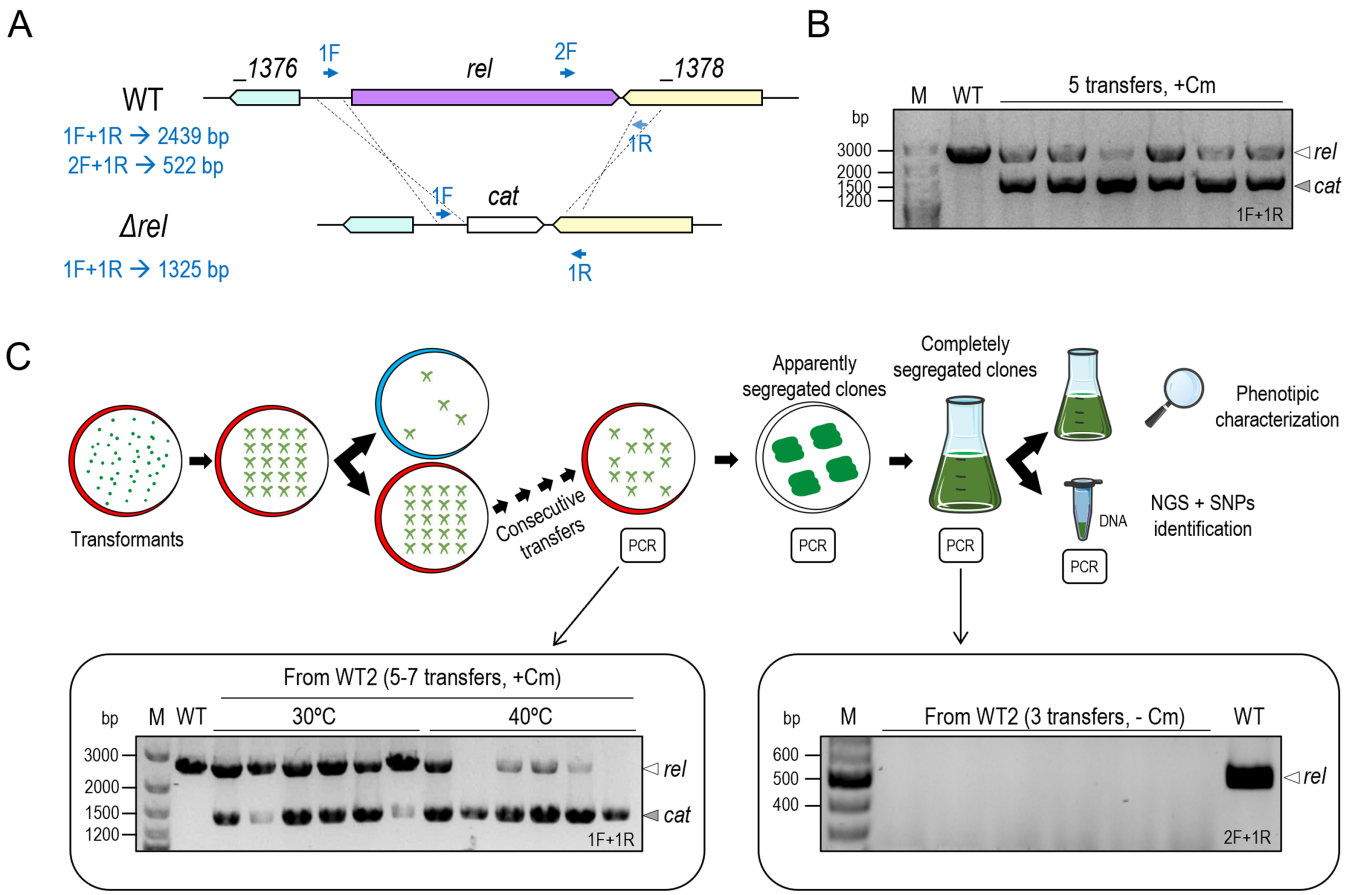
The *rel* gene is essential in *S. elongatus*. **(A)** Schematic representation of the *rel* locus in the *S. elongatus* chromosome and of the inactivation construct conferring chloramphenicol resistance. Primer positions for PCR analysis are depicted by blue arrows, with the size of expected products on the left. **(B)** Representative agarose gel showing PCR products from Amps Cm^2^ clones. **(C)** Workflow to obtain completely segregated *Δrel* clones. Selection of transformants and biomass transfers to allow allele segregation were performed on BG11 plates with chloramphenicol (red-edged plates; *bottom left*, representative gel). Ampicillin (blue-edged plate) was used to discard single recombinant clones. Apparently segregated clones were grown without chloramphenicol (white-edged plates), and retested for stable segregation of *Δrel*:*cat* alleles (*bottom right*, representative gel) before further analysis. The points at which PCR analyses were performed are indicated. The position of the relevant alleles are depicted by arrows on the right of gels. M, 100 bp size marker.

To construct a (p)ppGpp^0^ or Δ*rel* strain in *S. elongatus* we attempted allele replacement of the wild type *rel* sequences (Figure 1A), using plasmid pUC-ΔSynpcc7942_1377(rel)-Cm^R^ to follow exactly the strategy previously reported by Hood et al. (2016). To select double recombination in *S. elongatus*, the chloramphenicol-resistant transformant clones were checked in order to exclude ampicillin-resistant colonies carrying additional vector sequences. To identify clones homozygotic for the mutant allele (*Δrel:cat*), several consecutive transfers of Amp^S^ Cm^R^ clones onto Cm-containing plates were performed before PCR analysis aimed to detect both wild type (*rel*) and mutant (*Δrel:cat*) alleles. Despite the fact that most of the surviving clones grew poorly, significant amplification of the much larger *rel* allele took place in all cases (Figure 1B and Supplementary Figure S1A), indicating that, at least in our laboratory conditions, the *rel* gene is essential in *S. elongatus*.

### 3.2 Complete segregation of *rel* null alleles in *S. elongatus* requires prolonged subculturing under selective conditions

Our failure to completely inactivate the *rel* gene in *S. elongatus* was unexpected in the light of previous studies from other laboratories (Hood et al., 2016; Puszynska and O’Shea, 2017) and made us wonder on possible experimental differences, including culture conditions, incompletely segregated *S. elongatus* cultures or even the presence of suppressor mutations.

The inability to completely inactivate the *rel* gene in *S. elongatus* provided an opportunity to identify key targets controlled by (p)ppGpp in cyanobacteria under standard or relevant growth conditions. In this context, although *S. elongatus* cultures are routinely grown at 30–32 °C in most laboratories, including ours, they grow faster at high temperatures (Laws and McClellan, 2022; Ohbayashi et al., 2019; Rillema et al., 2021; Sun et al., 2023). Since temperature is an environmentally relevant condition reported to alter mutation rates (Berger et al., 2017; Chu et al., 2018; Riaz et al., 2022; Waldvogel and Pfenninger, 2021), we reasoned that performing the screenings at two different temperatures may increase the diversity of suppressor mutations allowing complete segregation of *Δrel:cat* alleles in *S. elongatus*.

The steps involved in the process of obtaining independent Δ*rel* clones are schematically illustrated and summarised in Figure 1C (top). Biomass from independently obtained Ap^S^ Cm^R^ clones was transferred onto selection plates and at least 30 double recombinant clones were incubated at either 30 °C or 40 °C and then subjected to additional transfers at the corresponding temperature. Given the temperature-dependent differences in growth rate, during the many weeks invested in the screening there was time for more consecutive transfers of the clones growing on plates at 40 °C than at 30 °C. After the first two consecutive transfers almost half of the clones did not grow, and PCR analysis of the surviving clones detected both *rel* and *Δrel:cat* alleles in all cases. The native *rel* alleles were undetectable in six different clones after 6–7 transfers (Figure 1C (bottom left) and Supplementary Figure S1B). To ensure that only completely segregated clones were processed for whole-genome sequencing, the six *Δrel* clones were inoculated into liquid medium without chloramphenicol before checking them again by PCR. All six clones retained the fully segregated *Δrel:cat* allele (Figure 1C (bottom right) and Supplementary Figure S1C). Since these clones came from the plates at 40 °C, the results suggested that the higher culture temperature favored segregation of *S. elongatus rel* null alleles.

### 3.3 *Bona fide* (p)ppGpp^0^ mutants of *S. elongatus* carry suppressor mutations, at genes involved in GTP biosynthesis or translation, that increase but do not restore viability

Whole-genome sequencing and comparison between the *S. elongatus* control and each of the 6 completely segregated Δ*rel* clones revealed genomic differences at one gene or two genes (Table 1, Search 1). One mutation was at *rpsB*, encoding ribosomal protein S2, the other six at *guaB3* or *guaA*, encoding purine biosynthesis enzymes inosine monophosphate dehydrogenase (IMPDH) and guanosine monophosphate synthase (GMPS), respectively. These three genes have been found essential in *S. elongatus* (Rubin et al., 2015), and thus the identified mutations must cause partial loss of function (see Discussion below). Furthermore, the only clone in which we identified mutations at two genes (*guaA* and *guaB3*) carried each of them in heterozygosity with the wild type allele, a result suggesting synthetic lethality between two genes involved in the same essential pathway.

**Table 1:** Summary of the mutations (SNPs/Indels) identified by NGS in Δ*rel* suppressor clones.

Gene ID (*synpcc7942*)	Product	Variation		Condition	Background	Search
*_0189*	GuaA	F338L	1,012 T > C	40 °C	WT2	1
*_0189* *_1831*	GuaA GuaB3	^h^F338L ^h^V281M	1,012 T > C 841G > A			
*_1831*	GuaB3	V227G	680 T > G			
		Q381L	1142A > T			
*_2530*	S2	H15Q, F16S	45CTT > AAG			
*_0189*	GuaA	M185V	555G > A	30 °C	WT2	2
*_0588*	PurT	V37fs	106_107dupGC / 110delT			
*_2530*	S2	H15Q, F16S	45CTT > AAG			
*_2530* *_0187*	S2 ORF0187	^h^H15Q, ^h^F16S ^h^S73I	45CTT > AAG 218G > T			
*_1831*	GuaB3	P147_V148ins8^◊^	417_440dup	40 °C		
*_2530*	S2	H15Q, F16S	45CTT > AAG			
*_2530*	S2	H15Q, F16S	45CTT > AAG	40 °C	WT1	

^h^Heterozygous mutation. ^◊^AlaGlyAlaAlaLysPheGlyPro.

Cultures from all six suppressor clones grew poorly and died rather fast, indicating that none of the suppressor mutations were able to restore viability to wild type levels. None of the corresponding cultures could be rescued from aliquots frozen after enough biomass was obtained for whole-genome sequencing, preventing any further characterization. Therefore, the results raised questions on the feasibility of obtaining a viable and relatively healthy mutant completely depleted of (p)ppGpp in *S. elongatus*, further supporting the essentiality of Rel function.

### 3.4 Suppressor screenings in two different laboratory strains of *S. elongatus*

The results presented so far provided a first snapshot of the functions and targets of (p)ppGpp in cyanobacteria, indicating that GTP levels and translation must be the main targets of (p)ppGpp control in this phylum. To gain additional insights into the issue, we performed additional suppressor searches.

**Figure 2:**
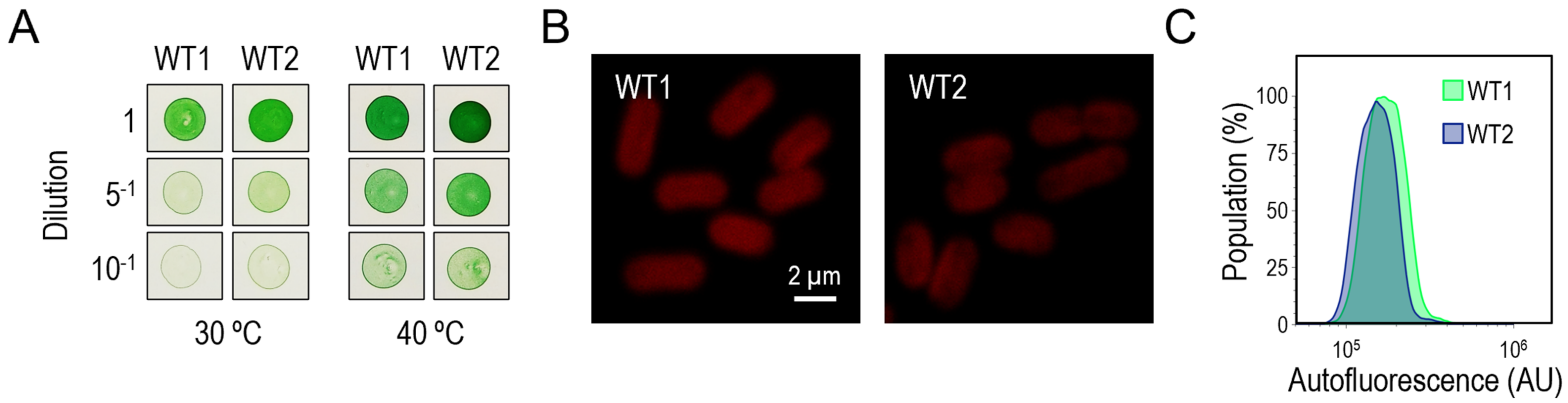
Phenotypic comparison of *S. elongatus* strains WT1 and WT2. **(A)** Drop-plate assay at either 30 °C or 40 °C (5 μL; initial OD^750^ = 0.5). **(B)** Representative confocal micrographs showing autofluorescence signal (ex640 nm). **(C)** Cell autofluorescence measured by flow cytometry (ex640 nm). AU, arbitrary units.

We reasoned that given the multiple regulatory roles of (p)ppGpp, the genetic background may affect the molecular details and outcome of suppressor searches aimed to identify intracellular targets. It is worth noting that subculturing wild type strains of *S. elongatus* under laboratory conditions contributes to phenotypic diversification and genomic changes (Adomako et al., 2022; Mendaña et al., 2025). In this context, several observations suggested that the cultures of *S. elongatus* wild type strain used in Search 1 (referred hereafter as WT2) differed genotypically from the ones (hereafter WT1) that were kept in the freezer. WT2 cultures grew slightly faster than WT1 (Figure 2A) and small differences in the autofluorescence signal of the cells, otherwise indistinguishable, were also observed by confocal microscopy and flow cytometry (Figures 2B,C), indicating that the genomes of these two strains have diverged in the last few years in our laboratory. In the absence of a precise hypothesis as to which genomic differences can be most relevant for the suppressor analysis, it appeared sensible to take advantage of the spontaneously occurring changes that separate WT1 and WT2, two closely related *S. elongatus* strains.

With the idea of increasing the total number and diversity of mutations suppressing Rel absence in *S. elongatus* we next used (WT1 and WT2) and two temperature conditions (30 °C and 40 °C) to carry out four parallel searches for clones with completely segregated *Δrel:cat* alleles. Almost 200 independent transformants were processed essentially as described above (Figure 1C). Remarkably, after withdrawing the antibiotic selection for a final confirmation, 34 out of the 54 apparently segregated clones recovered wild type *rel* sequences to very different extents (an example is shown in Supplementary Figure S2) and thus, only the 20 clones where *rel* sequences continued to be undetectable were considered *bona fide* (p)ppGpp^0^ strains. Those 20 clones (13 from WT1 and 7 from WT2), their wild type controls and one clone where *rel* sequences became detectable (from WT2), were then selected for phenotypic analysis and DNA extraction. All mutant clones, including the incompletely segregated one (hereafter Rel^+/−^), grew very slowly and lost viability in the following weeks.

### 3.5 Phenotypic diversity of *S. elongatus* (p)ppGpp^0^ cells

To phenotypically characterise the clones obtained in Search 2 before determining the identity of their putative suppressor mutation, we used experimental approaches that can be done in relatively short times and do not require very healthy cultures, including confocal microscopy, cell cytometry and growth plate assays.

**Figure 3:**
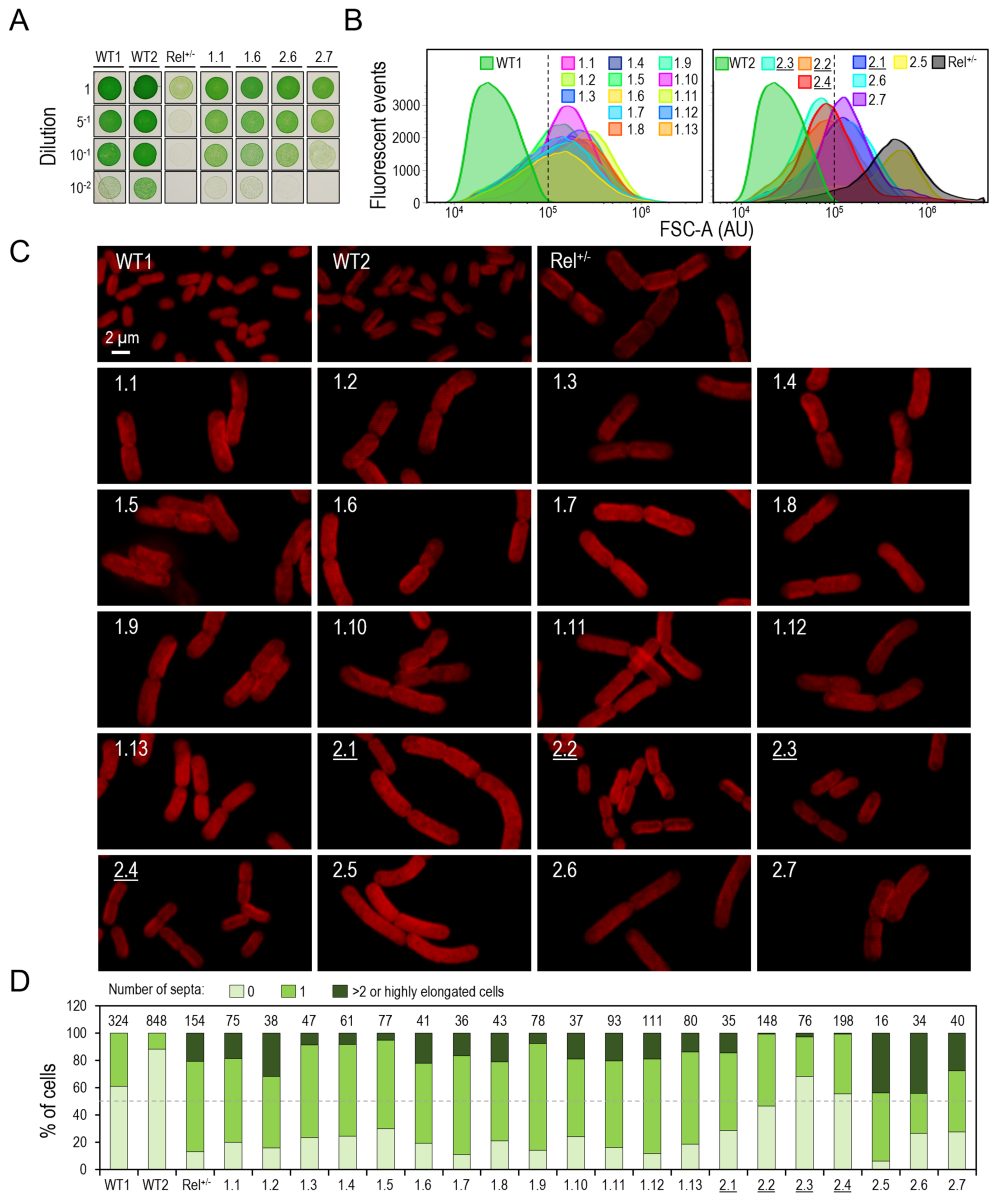
Phenotypes of completely and incompletely segregated *Δrel* clones. **(A)** Drop-plate assays of representative *Δrel* clones, Rel+/−, and control strains at 30 °C (5 μL; initial OD_750_ 0.5). **(B)** Cell size measured by FSC-A flow cytometry parameter. AU, arbitrary units. **(C)** Representative confocal micrographs showing autofluorescence signal (+20% brightness and contrast; ex640 nm). **(D)** Proportion of cells with 0, 1, or >2 septa/highly elongated, based on the number of cells indicated above. Samples are labeled as (origin).(clone), where 1.x and 2.x denote WT1 and WT2 backgrounds, respectively. Clones 2.1– to 2.4 are underlined to indicate that they do not carry mutation *rpsb* (clones 2.2–2.4) or that carry it in combination with another one (2.1).

As shown in Figure 3, the Rel^+/−^ clone showed the most severe phenotype for each of the cell or culture traits examined, and that was particularly evident when growth was compared (Figure 3A), indicating that complete segregation of the *Δrel:cat* allele implies suppression, to different extents, of phenotypic defects caused by the absence of (p)ppGpp. Significantly increased cell length and width were observed in all mutant cultures (Figures 3B,C and Supplementary Figure S3), with most of the clones exhibiting a high proportion of abnormally elongated cells and cells containing one or multiple septa, suggestive of impaired cell division (Figure 3D).

Two phenotypic groups emerged from the comparison of *bona fide* (p)ppGpp^0^ mutants. The most abundant one was phenotypically closer to the Rel^+/−^ strain, displaying a very high cell diameter, as measured by forward scatter area (FSC-A; median > 10^5^) (Figure 3B), and included all 13 derivatives from WT1 and 4 from WT2. The remaining clones (2.2, 2.3, and 2.4) were phenotypically closer to their WT2 control, showing a significantly lower proportion of cells containing septa (Figure 3D) and the lowest FSC-A values (median < 10^5^) (Figure 3B).

### 3.6 Mutations best suppressing (p)ppGpp^0^ defects target GTP biosynthesis

In close agreement with results from Search 1 indicating that *bona fide* (p)ppGpp^0^ mutants in *S. elongatus* carry suppressor mutations, whole-genome sequencing of clones from Search 2 revealed SNP differences with WT1 or WT2 controls in all but the incompletely segregated clone (Table 1). The mutations targeted the same genes identified in Search 1 (*guaA, guaB3, rpsB*) plus two additional ones: *purT,* encoding a phosphoribosylglycinamide formyltransferase of the *de novo* purine biosynthesis pathway, and *synpcc7942_0187*, encoding a protein of unknown function.

Once we identified the suppressor mutations corresponding to the clones analysed for differential cell features, it became obvious that the milder phenotypes corresponded to clones with mutations targeting purine biosynthesis: suppressor clones targeting GuaA (2.2), GuaB3 (2.4) or PurT (2.3). Therefore, amongst the mutations allowing certain level of growth in the absence of (p)ppGpp, those altering GTP levels are the most efficient suppressors.

### 3.7 Genomic mutations separating *S. elongatus* strains underline the regulatory complexity of (p)ppGpp signalling

Whole sequence analyses of all relevant clones revealed genomic differences between WT1, WT2, and the NCBI reference strain (Table 2). Comparison between WT1 and WT2 strains and the *S. elongatus* NCBI reference showed changes at eight genes, some of which would separate our *S. elongatus* cultures from those used in other laboratories. Differences between WT1 and WT2 were found at other five loci, and thus some or all of them would be responsible for the rather small phenotypic differences detected between our laboratory strains. Notwithstanding the possible contribution of the genomic changes identified here to the outcomes of suppressor searches with WT1 and WT2, the results call attention to the plasticity and regulatory complexity of (p)ppGpp signalling.

**Table 2:** Genomic variants (SNPs/Indels) identified by NGS in WT1 and WT2 strains compared with the NCBI reference genome (JACJTX01; *Synechococcus elongatus* PCC 7942 = FACHB-805).

Gene ID (*synpcc7942*)	Product	WT1	WT2
*_0329*	Methyltransferase domain-containing	L76fs	L76fs
*_0424*	PsbA1	^h^G276A ^h^M288L ^h^A294V ^h^K312R	^h^G276A ^h^M288L ^h^A294V ^h^K312R
*_0863*	Phosphotransacetylase family	R24G	R24G
*_1954*	CTP synthase	A294V	A294V
*_2114*	SasA	E3*	E3*
*_0052*	Methyltransferase domain-containing		V115N
*_1222*	TruB		W246*
*_1493*	MazG	V279F	
*_2070*	PilT	P124S	
*_2082* and *_2083*	^1^–338 from EF-G ^1^–12 from MarC-related		G > T
*_2373*	L-Ala-D/L-Glu epimerase	W98*	G27R

^h^Heterozygous mutation. ^1^Distances related to the ATG of the corresponding ORF.

## 4 Discussion

In *S. elongatus*, the levels of (p)ppGpp control global gene expression (Hood et al., 2016; Puszynska and O’Shea, 2017) during the alternation of light and darkness, the main environmental challenge of photosynthetic organisms. We show here that some levels of (p)ppGpp are also required for *S. elongatus* survival under standard laboratory growth conditions. Cultures retaining almost undetectable amounts of wild type *rel* alleles (Rel^+/−^) after prolonged culture were very sick, grew poorly and showed multiple cell defects that were ameliorated to different extents but not abolished by suppressor mutations. Although the rapid loss of viability affecting completely segregated Rel^−^ derivative strains prevented their biochemical characterization, other traits could be studied by microscopy, cell cytometry and growth assays, allowing comparison of the severity of relevant phenotypes amongst mutant strains.

Since gene essentiality often depends on genetic or environmental contexts and laboratory strains of *S. elongatus* are highly domesticated (Adomako et al., 2022), it is difficult to know whether (p)ppGpp^0^ strains reported from other laboratories were Rel^+/−^ or Rel^−^ derivatives carrying compensatory mutations. It is also worth noting that selection of spontaneous suppressor mutations during generation of homozygotic null mutants is favored by the need of prolonged subculturing due to the presence of several chromosome copies in *S. elongatus* (Watanabe, 2020). On the other hand, the essentiality of regulatory and multifunctional genes may be bypassed by mutations at other regulatory *loci* (North et al., 2025; Rosconi et al., 2022). In *S. elongatus* this is exemplified by the paradigmatic protein PII, involved in signalling of the carbon/nitrogen balance and energy in bacteria and plants (Bolay et al., 2021; Forchhammer et al., 2022; Forchhammer and Selim, 2020; Selim et al., 2020; Watzer et al., 2019). Functional studies involving PII deficient strains (Forchhammer and Tandeau de Marsac, 1995; Lee et al., 2000) were followed by the demonstration that the lack of PII had been bypassed by loss-of-function mutations targeting the interacting partner PipX, discovered years later (Chang et al., 2013; Espinosa et al., 2010, 2009; Sakamoto et al., 2021).

**Figure 4:**
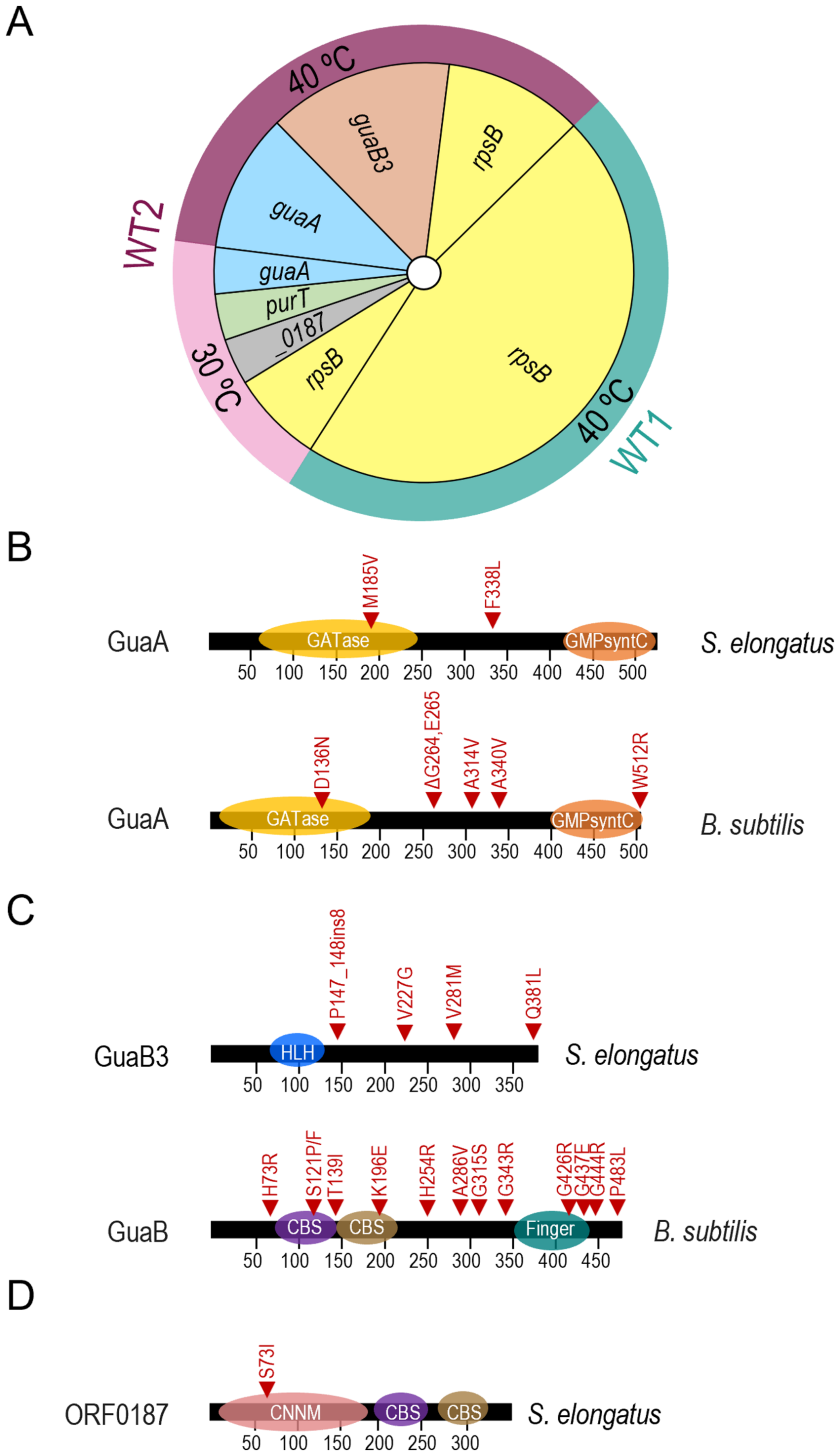
Suppressor mutations in *Δrel* clones. **(A)** Impact of temperature and genetic background on the genes targeted by suppressor mutations. Segments size represents the number of independent occurrences (minimum 1, maximum 13, see Table 1 for more details). **(B–D)** Schematic representation of domain organization at the indicated proteins from *S. elongatus* and/or *B. subtilis* showing the location (red arrowheads) of the suppressor mutations. GATase, glutamine amidotransferase class-I; GMPsyntC, GMP synthase C terminal domain; HLH, helix–loop–helix domain; CBS, cystathionine β-synthase domain; Finger, distinctive feature of the catalytic domain of IMDPHS; CNNM, cyclin M transmembrane N-terminal domain.

Given the regulatory complexity of (p)ppGpp signalling it is not surprising that both genetic and environmental factors affected the distribution of suppressor mutations (Figure 4A). Combined effects of higher mutation rate and faster growth at 40 °C may explain that the obtention of suppressor clones was more efficient at 40 °C (22 clones, 23 independent mutations) than at 30 °C (4 clones, 5 mutations). However, mutations at *purT* and *synpcc7942_0187* were found only at 30 °C, giving credit to the strategy of applying different environmental conditions to maximize the number of mutations and genes targeted in the suppressor analysis. Consistent with a clear dependence on the genetic context, all 13 independent suppressor clones from WT1 carried the same three nucleotide-long inversion (CTT to AAG) resulting in substitution of the consecutive residues 15His-16Phe for Gln-Ser at ribosomal protein S2 (hereafter mutation rpsB*). In contrast, only 5 out of 13 of the clones obtained from WT2 carried mutation at rpsB*. Since obtention of (p)ppGpp^0^ derivatives was not easier in WT1 than in WT2, the high recovery of rpsB* mutations in WT1 suggests that one or more of the genomic changes separating laboratory strains WT1 and WT2 favors selection of that particular mutation, mainly in detriment of mutations targeting purine biosynthesis.

The multiple occurrences of mutations targeting enzymes of the GTP biosynthesis pathway and the fact that those mutations rescued to a greater extent the effects of the absence of (p)ppGpp support the importance of this secondary messenger in the control of GTP levels in cyanobacteria. Furthermore, the similar phenotypes of all suppressor mutations targeting purine biosynthesis genes found in *S. elongatus* indicate similar metabolic consequences and it is thus tempting to propose that they all decrease GTP levels to similar extent. The mutation at the non-essential gene *purT* from the *de novo* pathway was a frameshift, suggestive of complete loss of function. In contrast, the suppressor mutations at the essential genes *guaA or guaB3* were single amino acid substitutions (or, in one case, a small in-frame insertion), suggesting decrease in the activity of the corresponding gene products, as in equivalent suppressor searches in *B. subtilis* (Kriel et al., 2012; Osaka et al., 2020). The distribution of suppressor mutations along the protein sequence found at GuaA and GuaB(3) are shown in Figures 4B,C. The location of the corresponding *S. elongatus* residues targeted on the recently determined GuaB3 structure (Hernández-Gómez et al., 2023) is shown in Supplementary Figure S4.

In bacteria guanine nucleotides ((p)ppGpp and GTP/GDP) modulate the activities of many accessory proteins and translation factors (Cancino-Diaz et al., 2023; Hauryliuk et al., 2015). In firmicutes, high levels of GTP kill the cells and most of the known suppressor mutations of (p)ppGpp^0^ mutants target genes involved in GTP biosynthesis (Kriel et al., 2012; Osaka et al., 2020) (Supplementary Table S2). In cyanobacteria, as in gram positives, ribosome activity should be highly dependent on GTP levels, since GTP is also the initiating nucleotide for rRNA transcription in *S. elongatus* (Kumano et al., 1986). Our results indicate that in the absence of (p)ppGpp rpsB* compensate the excess of GTP by altering the structure of the ribosome to decrease translation by a yet unknown mechanism. It is worth noting that on the one hand, the residues targeted by the rpsB* mutation appear relevant for the interaction with S1 at the ribosome (Byrgazov et al., 2015; Webster et al., 2024). On the other hand, S2 from *B. subtilis* interacts with HPF (Hibernation-Promoting Factor) (Beckert et al., 2017), a protein regulating ribosomal status in response to (p)ppGpp (Feaga et al., 2020; Hood et al., 2016; Nagarajan et al., 2025). That interaction is induced by (p)ppGpp and results in ribosome dimerization and protection of small subunit degradation (Feaga et al., 2020). It is also worth noting that HPF shows distinct structural features in cyanobacteria (Contreras et al., 2018; Neira et al., 2018) and that ribosomal structures are not yet available to clarify whether in *S. elongatus* the S2 variant compensates for lack of (p)ppGpp by affecting interactions with S1, HPF and/or other translation related factors.

While the role of (p)ppGpp in the control of GTP levels appears to be a hallmark of both firmicutes and cyanobacteria, the control of key regulators and enzymes appear to be different. The transcriptional regulator CodY that senses GTP (Shivers and Sonenshein, 2004) and controls expression of guanine biosynthesis genes is absent in cyanobacteria and key enzymes such as IMPDH, which is illustrated in Figure 4C (GuaB/GuaB3) have different structural and regulatory features (Hernández-Gómez et al., 2023). Not surprisingly, this study identified an unknown protein (ORF0187) as a putative(p)ppGpp target in cyanobacteria. It is worth noting that, although the mutation at the gene *synpcc7942_0187*, classified as beneficial (Rubin et al., 2015), was found in combination with rpsB*, both mutations were found in heterozygosity. Since in clones with a pair of incompletely segregated mutations both tend to contribute to the phenotype, it is likely that the mutation at *synpcc7942_0187* would also contribute to suppress Rel deficiency.

Remarkably, ORF0187 is annotated in some databases as HlyC/CorC family transporter or TlyC even though the CorC domain is absent. ORF0187, a membrane protein that appears to be poorly expressed during growth under standard laboratory conditions in *S. elongatus* (Perrin et al., 2025) has a unique combination of conserved domains (Figure 4D). A cyclin M transmembrane N-terminal domain (CNNM) is followed by two cystathionine β-synthase domains (CBS) or CBS pair forming a so-called Bateman domain (Bateman, 1997). CBS pairs are present in canonical IMPDH enzymes such as bacterial GuaB but not in cyanobacterial GuaB3 and thus its presence in ORF0187 is highly intriguing. Given the involvement of CBS pairs in nucleotide signaling, it is tempting to propose a signaling role for ORF0187 in the context of nucleotide homeostasis. The mutation identified here may impair protein function by affecting dimerization of the transmembrane domain.

Genetic searches for (p)ppGpp^0^ suppressors in different bacteria identified different key targets. According to the literature on the type and numbers of independent mutations previously reported (summarised in Supplementary Table S2), the guanine biosynthesis pathway appears to be the main target in both firmicutes and cyanobacteria [(Kriel et al., 2012; Osaka et al., 2020); this work]. Transcription (RNA polymerase subunits) would be the main target in proteobacteria, and also important in firmicutes (Hernandez and Cashel, 1995; Laurie et al., 2003; Murphy and Cashel, 2003; Osaka et al., 2020; Perrier et al., 2025; Szalewska-Palasz et al., 2007; Wells and Long, 2003). Remarkably, amongst the several hundreds of (p)ppGpp^0^ suppressor mutations already reported from different bacteria, those targeting translation have only been found in cyanobacteria (S2 protein, this work), raising questions on the molecular basis involved.

In summary, we have shown here that some levels of (p)ppGpp are required for *S. elongatus* viability under standard laboratory growth conditions and that obtention (p)ppGpp^0^ derivatives requires suppressor mutations. These mutations identified GTP homeostasis and translation as the main targets of (p)ppGpp signalling in cyanobacteria as well as a novel membrane protein predicted to signal nucleotide homeostasis. Our results provide useful insights into the functions and targets of (p)ppGpp in this important phylum while calling attention to the significant impact that mutations emerging in the laboratory, in addition to other environmental differences and culture conditions, may have on genetic screenings. While this calls for caution in interpretation of results, it also underlines the power of genetic approaches to identify the relevant targets and processes targeted by promiscuous and versatile cellular compounds such as (p)ppGpp.

## Data Availability

The raw reads from the whole genome sequencing of rel mutant suppressor pools are available on the European Nucleotide Archive (ENA; https://www.ebi.ac.uk/ena/browser/view/PRJEB110973) under accession numbers SAMEA122076247, SAMEA122076248, SAMEA122076249, SAMEA122076250, SAMEA12207651, SAMEA122076252, SAMEA122076253, SAMEA122076254, SAMEA122076255, and SAMEA122076256 from the BioProject PRJEB110973.

## Footnotes

1
https://floreada.io

## References

[ref1] Adomako M. Ernst D. Simkovsky R. Chao Y.-Y. Wang J. Fang M. . (2022). Comparative genomics of *Synechococcus elongatus* explains the phenotypic diversity of the strains. MBio 13:e0086222. doi: 10.1128/mbio.00862-22, 35475644 PMC9239245

[ref2] Akinyanju J. Smith R. J. (1979). Accumulation of ppGpp and pppGpp during nitrogen deprivation of the cyanophyte *Anabaena cylindrica*. FEBS Lett. 107, 173–176. doi: 10.1016/0014-5793(79)80489-5, 115718

[ref3] Anderson S. E. Vadia S. E. McKelvy J. Levin P. A. (2023). The transcription factor DksA exerts opposing effects on cell division depending on the presence of ppGpp. MBio:14. doi: 10.1128/mbio.02425-23 PMC10746185 37882534

[ref4] Atkinson G. C. Tenson T. Hauryliuk V. (2011). The RelA/SpoT homolog (RSH) superfamily: distribution and functional evolution of ppGpp Synthetases and hydrolases across the tree of life. PLoS One 6:e23479. doi: 10.1371/journal.pone.0023479, 21858139 PMC3153485

[ref5] Bange G. Brodersen D. E. Liuzzi A. Steinchen W. (2021). Two P or not two P: understanding regulation by the bacterial second messengers (p)ppGpp. Ann. Rev. Microbiol. 75, 383–406. doi: 10.1146/annurev-micro-042621-122343 34343020

[ref6] Bateman A. (1997). The structure of a domain common to archaebacteria and the homocystinuria disease protein. Trends Biochem. Sci. 22, 12–13. doi: 10.1016/S0968-0004(96)30046-7, 9020585

[ref7] Beckert B. Abdelshahid M. Schäfer H. Steinchen W. Arenz S. Berninghausen O. . (2017). Structure of the *Bacillus subtilis* hibernating 100S ribosome reveals the basis for 70S dimerization. EMBO J. 36, 2061–2072. doi: 10.15252/embj.201696189, 28468753 PMC5509997

[ref8] Bennison D. J. Nakamoto J. A. Craggs T. D. Milón P. Rafferty J. B. Corrigan R. M. (2021). The stringent response inhibits 70S ribosome formation in *Staphylococcus aureus* by impeding GTPase-ribosome interactions. MBio 12:e0267921. doi: 10.1128/mBio.02679-21, 34749534 PMC8579695

[ref9] Berger D. Stångberg J. Grieshop K. Martinossi-Allibert I. Arnqvist G. (2017). Temperature effects on life-history trade-offs, germline maintenance and mutation rate under simulated climate warming. Proc. R. Soc. B Biol. Sci. 284:20171721. doi: 10.1098/rspb.2017.1721, 29118134 PMC5698646

[ref10] Bolay P. Rozbeh R. Muro-Pastor M. I. Timm S. Hagemann M. Florencio F. J. . (2021). The novel PII -interacting protein PirA controls flux into the cyanobacterial ornithine-Ammonia cycle. MBio 12:21. doi: 10.1128/mBio.00229-21 PMC8092223 33758091

[ref11] Byrgazov K. Grishkovskaya I. Arenz S. Coudevylle N. Temmel H. Wilson D. N. . (2015). Structural basis for the interaction of protein S1 with the *Escherichia coli* ribosome. Nucleic Acids Res. 43, 661–673. doi: 10.1093/nar/gku1314 25510494 PMC4288201

[ref12] Cancino-Diaz M. E. Guerrero-Barajas C. Betanzos-Cabrera G. Cancino-Diaz J. C. (2023). Nucleotides as bacterial second messengers. Molecules 28:7996. doi: 10.3390/molecules28247996, 38138485 PMC10745434

[ref13] Cashel M. Gallant J. (1969). Two compounds implicated in the function of the *RC* gene of *Escherichia coli*. Nature 221, 838–841. doi: 10.1038/221838a0, 4885263

[ref14] Chang Y. Takatani N. Aichi M. Maeda S. Omata T. (2013). Evaluation of the effects of PII deficiency and the toxicity of PipX on growth characteristics of the PII-less mutant of the cyanobacterium *Synechococcus elongatus*. Plant Cell Physiol. 54, 1504–1514. doi: 10.1093/pcp/pct092 23811238

[ref15] Chi J.-T. Zhou P. (2023). From magic spot ppGpp to MESH1: stringent response from bacteria to metazoa. PLoS Pathog. 19:e1011105. doi: 10.1371/journal.ppat.1011105, 36730138 PMC9894426

[ref16] Chu X.-L. Zhang B.-W. Zhang Q.-G. Zhu B.-R. Lin K. Zhang D.-Y. (2018). Temperature responses of mutation rate and mutational spectrum in an *Escherichia coli* strain and the correlation with metabolic rate. BMC Evol. Biol. 18:126. doi: 10.1186/s12862-018-1252-8, 30157765 PMC6116381

[ref17] Contreras L. M. Sevilla P. Cámara-Artigas A. Hernández-Cifre J. G. Rizzuti B. Florencio F. J. . (2018). The cyanobacterial ribosomal-associated protein LrtA from *Synechocystis* sp. PCC 6803 is an Oligomeric protein in solution with chameleonic sequence properties. Int. J. Mol. Sci. 19:1857. doi: 10.3390/ijms19071857, 29937518 PMC6073757

[ref18] Dworkin J. (2023). Understanding the stringent response: experimental context matters. MBio 2023:14. doi: 10.1128/mbio.03404-22 PMC9973329 36625599

[ref19] Espinosa J. Castells M. A. Laichoubi K. B. Contreras A. (2009). Mutations at *pipX* suppress lethality of P_II_ -deficient mutants of *Synechococcus elongatus* PCC 7942. J. Bacteriol. 191, 4863–4869. doi: 10.1128/JB.00557-09, 19482921 PMC2715732

[ref20] Espinosa J. Castells M. A. Laichoubi K. B. Forchhammer K. Contreras A. (2010). Effects of spontaneous mutations in PipX functions and regulatory complexes on the cyanobacterium *Synechococcus elongatus* strain PCC 7942. Microbiology 156, 1517–1526. doi: 10.1099/mic.0.037309-0 20110304

[ref21] Feaga H. A. Kopylov M. Kim J. K. Jovanovic M. Dworkin J. (2020). Ribosome dimerization protects the small subunit. J. Bacteriol. 202:20. doi: 10.1128/JB.00009-20 PMC7186458 32123037

[ref22] Fernández-Coll L. Cashel M. (2020). Possible roles for basal levels of (p)ppGpp: growth efficiency vs. surviving stress. Front. Microbiol. 11:592718. doi: 10.3389/fmicb.2020.592718 33162969 PMC7581894

[ref23] Field B. (2018). Green magic: regulation of the chloroplast stress response by (p)ppGpp in plants and algae. J. Exp. Bot. 69, 2797–2807. doi: 10.1093/jxb/erx485, 29281108

[ref24] Forchhammer K. Selim K. A. (2020). Carbon/nitrogen homeostasis control in cyanobacteria. FEMS Microbiol. Rev. 44, 33–53. doi: 10.1093/femsre/fuz025, 31617886 PMC8042125

[ref25] Forchhammer K. Selim K. A. Huergo L. F. (2022). New views on PII signaling: from nitrogen sensing to global metabolic control. Trends Microbiol. 30, 722–735. doi: 10.1016/j.tim.2021.12.014, 35067429

[ref26] Forchhammer K. Tandeau de Marsac N. (1995). Functional analysis of the phosphoprotein PII (*glnB* gene product) in the cyanobacterium *Synechococcus* sp. strain PCC 7942. J. Bacteriol. 177, 2033–2040. doi: 10.1128/jb.177.8.2033-2040.1995, 7721695 PMC176846

[ref27] Friga G. M. Borbely G. Farkas G. L. (1981). Accumulation of guanosine tetraphosphate (ppGpp) under nitrogen starvation in *Anacystis nidulans*, a cyanobacterium. Arch. Microbiol. 129, 341–343. doi: 10.1007/BF00406458, 6793016

[ref28] Gourse R. L. Chen A. Y. Gopalkrishnan S. Sanchez-Vazquez P. Myers A. Ross W. (2018). Transcriptional responses to ppGpp and DksA. Ann. Rev. Microbiol. 72, 163–184. doi: 10.1146/annurev-micro-090817-062444, 30200857 PMC6586590

[ref29] Hauryliuk V. Atkinson G. C. Murakami K. S. Tenson T. Gerdes K. (2015). Recent functional insights into the role of (p)ppGpp in bacterial physiology. Nat. Rev. Microbiol. 13, 298–309. doi: 10.1038/nrmicro3448, 25853779 PMC4659695

[ref30] Hernandez J. V. Cashel M. (1995). Changes in conserved region 3 of *Escherichia coli* σ70 mediate ppGpp-dependent functions in vivo. J. Mol. Biol. 252, 536–549. doi: 10.1006/jmbi.1995.0518, 7563072

[ref31] Hernández-Gómez A. Irisarri I. Fernández-Justel D. Peláez R. Jiménez A. Revuelta J. L. . (2023). GuaB3, an overlooked enzyme in cyanobacteria’s toolbox that sheds light on IMP dehydrogenase evolution. Structure 31, 1526–1534.e4. doi: 10.1016/j.str.2023.09.014, 37875114

[ref32] Hidese R. Ohbayashi R. Kato Y. Matsuda M. Tanaka K. Imamura S. . (2023). ppGpp accumulation reduces the expression of the global nitrogen homeostasis-modulating NtcA regulon by affecting 2-oxoglutarate levels. Commun. Biol. 6:1285. doi: 10.1038/s42003-023-05632-1, 38145988 PMC10749895

[ref33] Holtman C. K. Chen Y. Sandoval P. Gonzales A. Nalty M. S. Thomas T. L. . (2005). High-throughput functional analysis of the *Synechococcus elongatus* PCC 7942 genome. DNA Res. 12, 103–115. doi: 10.1093/dnares/12.2.103, 16303742

[ref34] Hood R. D. Higgins S. A. Flamholz A. Nichols R. J. Savage D. F. (2016). The stringent response regulates adaptation to darkness in the cyanobacterium *Synechococcus elongatus*. Proc. Natl. Acad. Sci. 113, E4867–E4876. doi: 10.1073/pnas.1524915113, 27486247 PMC4995992

[ref35] Horvatek P. Salzer A. Hanna A. M. F. Gratani F. L. Keinhörster D. Korn N. . (2020). Inducible expression of (pp)pGpp synthetases in *Staphylococcus aureus* is associated with activation of stress response genes. PLoS Genet. 16:e1009282. doi: 10.1371/journal.pgen.1009282, 33378356 PMC7802963

[ref36] Hou F. Ke Z. Xu Y. Wang Y. Zhu G. Gao H. . (2023). Systematic large fragment deletions in the genome of *Synechococcus elongatus* and the consequent changes in transcriptomic profiles. Genes (Basel) 14:1091. doi: 10.3390/genes14051091, 37239451 PMC10217888

[ref37] Irving S. E. Choudhury N. R. Corrigan R. M. (2021). The stringent response and physiological roles of (pp)pGpp in bacteria. Nat. Rev. Microbiol. 19, 256–271. doi: 10.1038/s41579-020-00470-y, 33149273

[ref38] Irving S. E. Corrigan R. M. (2018). Triggering the stringent response: signals responsible for activating (p)ppGpp synthesis in bacteria. Microbiology 164, 268–276. doi: 10.1099/mic.0.000621, 29493495

[ref39] Ito D. Ihara Y. Nishihara H. Masuda S. (2017). Phylogenetic analysis of proteins involved in the stringent response in plant cells. J. Plant Res. 130, 625–634. doi: 10.1007/s10265-017-0922-8, 28303404

[ref40] Jones P. G. Cashel M. Glaser G. Neidhardt F. C. (1992). Function of a relaxed-like state following temperature downshifts in *Escherichia coli*. J. Bacteriol. 174, 3903–3914. doi: 10.1128/jb.174.12.3903-3914.1992, 1597413 PMC206098

[ref41] Kessler J. R. Cobe B. L. Richards G. R. (2017). Stringent response regulators contribute to recovery from glucose phosphate stress in *Escherichia coli*. Appl. Environ. Microbiol. 83:17. doi: 10.1128/AEM.01636-17, 28986375 PMC5717215

[ref42] Kriel A. Bittner A. N. Kim S. H. Liu K. Tehranchi A. K. Zou W. Y. . (2012). Direct regulation of GTP homeostasis by (p)ppGpp: a critical component of viability and stress resistance. Mol. Cell 48, 231–241. doi: 10.1016/j.molcel.2012.08.009, 22981860 PMC3483369

[ref43] Kumano M. Tomioka N. Shinozaki K. Sugiura M. (1986). Analysis of the promoter region in the *rrnA* operon from a blue-green alga, *Anacystis nidulans* 6301. Mol. Gen. Genet. 202, 173–178. doi: 10.1007/BF00331633

[ref44] Labella J. I. Cantos R. Salinas P. Espinosa J. Contreras A. (2020a). Distinctive features of PipX, a unique signaling protein of Cyanobacteria. Life 10:79. doi: 10.3390/life10060079, 32481703 PMC7344720

[ref45] Labella J. I. Llop A. Contreras A. (2020b). The default cyanobacterial linked genome: an interactive platform based on cyanobacterial linkage networks to assist functional genomics. FEBS Lett. 594, 1661–1674. doi: 10.1002/1873-3468.13775, 32233038

[ref46] Laurie A. D. Bernardo L. M. D. Sze C. C. Skärfstad E. Szalewska-Palasz A. Nyström T. . (2003). The role of the alarmone (p)ppGpp in ςN competition for core RNA polymerase. J. Biol. Chem. 278, 1494–1503. doi: 10.1074/jbc.M209268200 12421818

[ref47] Laws E. A. McClellan S. A. (2022). Interactive effects of CO_2_, temperature, irradiance, and nutrient limitation on the growth and physiology of the marine cyanobacterium *Synechococcus* (Cyanophyceae). J. Phycol. 58, 703–718. doi: 10.1111/jpy.13278, 35830205 PMC9805005

[ref48] Lee H. Flores E. Forchhammer K. Herrero A. Tandeau de Marsac N. (2000). Phosphorylation of the signal transducer PII protein and an additional effector are required for the PII -mediated regulation of nitrate and nitrite uptake in the cyanobacterium *Synechococcus* sp. PCC 7942. Eur. J. Biochem. 267, 591–600. doi: 10.1046/j.1432-1327.2000.01043.x, 10632730

[ref49] Levdikov V. M. Blagova E. Young V. L. Belitsky B. R. Lebedev A. Sonenshein A. L. . (2017). Structure of the branched-chain amino acid and GTP-sensing global regulator, CodY, from *Bacillus subtilis*. J. Biol. Chem. 292, 2714–2728. doi: 10.1074/jbc.M116.754309, 28011634 PMC5314169

[ref50] Liu K. Bittner A. N. Wang J. D. (2015). Diversity in (p)ppGpp metabolism and effectors. Curr. Opin. Microbiol. 24, 72–79. doi: 10.1016/j.mib.2015.01.012, 25636134 PMC4380541

[ref51] Llop A. Labella J. I. Borisova M. Forchhammer K. Selim K. A. Contreras A. (2023). Pleiotropic effects of PipX, PipY, or RelQ overexpression on growth, cell size, photosynthesis, and polyphosphate accumulation in the cyanobacterium *Synechococcus elongatus* PCC7942. Front. Microbiol. 14:1141775. doi: 10.3389/fmicb.2023.1141775, 37007489 PMC10060972

[ref52] Mendaña A. Santos-Merino M. Gutiérrez-Lanza R. Domínguez-Quintero M. Medina-Méndez J. M. González-Guerra A. . (2025). Mutations in the circadian cycle drive adaptive plasticity in cyanobacteria. Proc. Natl. Acad. Sci. 122:e2506928122. doi: 10.1073/pnas.2506928122, 40901874 PMC12435244

[ref53] Murphy H. Cashel M. (2003). Isolation of RNA polymerase suppressors of a (p)ppGpp deficiency. Methods Enzymol. 2003, 596–601. doi: 10.1016/S0076-6879(03)71044-1 14712731

[ref54] Nagarajan S. N. Rosenthal A. Dworkin J. (2025). (p)ppGpp-dependent activation of gene expression during nutrient limitation. MBio 16:e0128825. doi: 10.1128/mbio.01288-25, 40823838 PMC12421865

[ref55] Neira J. L. Giudici A. M. Hornos F. Arbe A. Rizzuti B. (2018). The C terminus of the ribosomal-associated protein LrtA is an intrinsically disordered oligomer. Int. J. Mol. Sci. 19:3902. doi: 10.3390/ijms19123902 30563168 PMC6321479

[ref56] North H. Hydorn M. Dworkin J. Fiebig A. Crosson S. (2025). Disrupting NtrC function reveals unexpected robustness in a central cell cycle regulatory network. MBio 16:25. doi: 10.1128/mbio.01962-25, 40823827 PMC12421884

[ref57] Ohbayashi R. Nakamachi A. Hatakeyama T. S. Watanabe S. Kanesaki Y. Chibazakura T. . (2019). Coordination of Polyploid chromosome replication with cell size and growth in a cyanobacterium. MBio 10:19. doi: 10.1128/mBio.00510-19 PMC6478999 31015323

[ref58] Osaka N. Kanesaki Y. Watanabe M. Watanabe S. Chibazakura T. Takada H. . (2020). Novel (p)ppGpp ^0^ suppressor mutations reveal an unexpected link between methionine catabolism and GTP synthesis in *Bacillus subtilis*. Mol. Microbiol. 113, 1155–1169. doi: 10.1111/mmi.14484, 32052499

[ref59] Parshin A. Shiver A. L. Lee J. Ozerova M. Schneidman-Duhovny D. Gross C. A. . (2015). DksA regulates RNA polymerase in *Escherichia coli* through a network of interactions in the secondary channel that includes sequence insertion 1. Proc. Natl. Acad. Sci. 112, E6862–E6871. doi: 10.1073/pnas.1521365112, 26604313 PMC4687573

[ref60] Paul B. J. Barker M. M. Ross W. Schneider D. A. Webb C. Foster J. W. . (2004). DksA: a critical component of the transcription initiation machinery that potentiates the regulation of rRNA promoters by ppGpp and the initiating NTP. Cell 118, 311–322. doi: 10.1016/j.cell.2004.07.009, 15294157

[ref61] Pausch P. Steinchen W. Wieland M. Klaus T. Freibert S.-A. Altegoer F. . (2018). Structural basis for (p)ppGpp-mediated inhibition of the GTPase RbgA. J. Biol. Chem. 293, 19699–19709. doi: 10.1074/jbc.RA118.003070, 30366986 PMC6314131

[ref62] Perrier A. Budin-Verneuil A. Hallez R. (2025). Functional interplay between (p)ppGpp and RNAP in *Acinetobacter baumannii*. PLoS Pathog. 21:e1013795. doi: 10.1371/journal.ppat.1013795, 41411374 PMC12742793

[ref63] Perrin A. J. Dowson M. Davis K. Nam O. Dowle A. A. Calder G. . (2025). CyanoTag: discovery of protein function facilitated by high-throughput endogenous tagging in a photosynthetic prokaryote. Sci. Adv. 11:eadp6599. doi: 10.1126/sciadv.adp6599, 39919180 PMC11804935

[ref64] Potrykus K. Bryszkowska K. Gąsior F. Klasa W. (2026). (p)ppGpp: the magic goes on. Microbiol. Mol. Biol. Rev. doi: 10.1128/mmbr.00413-25 PMC13296368 42053290

[ref65] Potrykus K. Murphy H. Philippe N. Cashel M. (2011). ppGpp is the major source of growth rate control in *E. coli*. Environ. Microbiol. 13, 563–575. doi: 10.1111/j.1462-2920.2010.02357.x 20946586 PMC4556285

[ref66] Puszynska A. M. O’Shea E. K. (2017). ppGpp controls global gene expression in light and in darkness in *S. elongatus*. Cell Rep. 21, 3155–3165. doi: 10.1016/j.celrep.2017.11.067, 29241543

[ref67] Riaz S. Jiang Y. Xiao M. You D. Klepacz-Smółka A. Rasul F. . (2022). Generation of miniploid cells and improved natural transformation procedure for a model cyanobacterium *Synechococcus elongatus* PCC 7942. Front. Microbiol. 13:959043. doi: 10.3389/fmicb.2022.959043, 35958137 PMC9360974

[ref68] Rillema R. Hoang Y. MacCready J. S. Vecchiarelli A. G. (2021). Carboxysome Mispositioning alters growth, morphology, and rubisco level of the cyanobacterium *Synechococcus elongatus* PCC 7942. MBio 12:20. doi: 10.1128/mBio.02696-20, 34340540 PMC8406218

[ref69] Rippka R. Deruelles J. Waterbury J. B. Herdman M. Stanier R. Y. (1979). Generic assignments, strain histories and properties of pure cultures of Cyanobacteria. Microbiology 111, 1–61. doi: 10.1099/00221287-111-1-1

[ref70] Romand S. Abdelkefi H. Lecampion C. Belaroussi M. Dussenne M. Ksas B. . (2022). A guanosine tetraphosphate (ppGpp) mediated brake on photosynthesis is required for acclimation to nitrogen limitation in *Arabidopsis*. eLife 11:e75041. doi: 10.7554/eLife.75041 35156611 PMC8887892

[ref71] Rosconi F. Rudmann E. Li J. Surujon D. Anthony J. Frank M. . (2022). A bacterial pan-genome makes gene essentiality strain-dependent and evolvable. Nat. Microbiol. 7, 1580–1592. doi: 10.1038/s41564-022-01208-7, 36097170 PMC9519441

[ref72] Ross W. Sanchez-Vazquez P. Chen A. Y. Lee J.-H. Burgos H. L. Gourse R. L. (2016). ppGpp binding to a site at the RNAP-DksA interface accounts for its dramatic effects on transcription initiation during the stringent response. Mol. Cell 62, 811–823. doi: 10.1016/j.molcel.2016.04.029, 27237053 PMC4912440

[ref73] Ross W. Vrentas C. E. Sanchez-Vazquez P. Gaal T. Gourse R. L. (2013). The magic spot: a ppGpp binding site on *E. coli* RNA polymerase responsible for regulation of transcription initiation. Mol. Cell 50, 420–429. doi: 10.1016/j.molcel.2013.03.021, 23623682 PMC3654024

[ref74] Rubin B. E. Wetmore K. M. Price M. N. Diamond S. Shultzaberger R. K. Lowe L. C. . (2015). The essential gene set of a photosynthetic organism. Proc. Natl. Acad. Sci. 112, E6634–E6643. doi: 10.1073/pnas.1519220112, 26508635 PMC4672817

[ref75] Ruwe M. Persicke M. Busche T. Müller B. Kalinowski J. (2019). Physiology and transcriptional analysis of (p)ppGpp-related regulatory effects in *Corynebacterium glutamicum*. Front. Microbiol. 10:2769. doi: 10.3389/fmicb.2019.02769, 31849906 PMC6892785

[ref76] Sakamoto T. Takatani N. Sonoike K. Jimbo H. Nishiyama Y. Omata T. (2021). Dissection of the mechanisms of growth inhibition resulting from loss of the PII protein in the cyanobacterium *Synechococcus elongatus* PCC 7942. Plant Cell Physiol. 62, 721–731. doi: 10.1093/pcp/pcab030, 33650637 PMC8474142

[ref77] Sanchez-Vazquez P. Dewey C. N. Kitten N. Ross W. Gourse R. L. (2019). Genome-wide effects on *Escherichia coli* transcription from ppGpp binding to its two sites on RNA polymerase. Proc. Natl. Acad. Sci. 116, 8310–8319. doi: 10.1073/pnas.1819682116, 30971496 PMC6486775

[ref78] Selim K. A. Ermilova E. Forchhammer K. (2020). From cyanobacteria to Archaeplastida: new evolutionary insights into PII signalling in the plant kingdom. New Phytol. 227, 722–731. doi: 10.1111/nph.16492, 32077495

[ref79] Shivers R. P. Sonenshein A. L. (2004). Activation of the *Bacillus subtilis* global regulator CodY by direct interaction with branched-chain amino acids. Mol. Microbiol. 53, 599–611. doi: 10.1111/j.1365-2958.2004.04135.x 15228537

[ref80] Sun H. Luan G. Ma Y. Lou W. Chen R. Feng D. . (2023). Engineered hypermutation adapts cyanobacterial photosynthesis to combined high light and high temperature stress. Nat. Commun. 14:1238. doi: 10.1038/s41467-023-36964-5, 36871084 PMC9985602

[ref81] Surányi G. Korcz A. Pálfi Z. Borbély G. (1987). Effects of light deprivation on RNA synthesis, accumulation of guanosine 3′(2′)-diphosphate 5′-diphosphate, and protein synthesis in heat-shocked *Synechococcus* sp. strain PCC 6301, a cyanobacterium. J. Bacteriol. 169, 632–639. doi: 10.1128/jb.169.2.632-639.1987, 2433265 PMC211825

[ref82] Szalewska-Palasz A. Johansson L. U. M. Bernardo L. M. D. Skärfstad E. Stec E. Brännström K. . (2007). Properties of RNA polymerase bypass mutants. J. Biol. Chem. 282, 18046–18056. doi: 10.1074/jbc.M610181200, 17456470

[ref83] Taton A. Erikson C. Yang Y. Rubin B. E. Rifkin S. A. Golden J. W. . (2020). The circadian clock and darkness control natural competence in cyanobacteria. Nat. Commun. 11:1688. doi: 10.1038/s41467-020-15384-9, 32245943 PMC7125226

[ref84] Tremiño L. Llop A. Rubio V. Contreras A. (2022). The conserved family of the pyridoxal phosphate-binding protein (PLPBP) and its cyanobacterial paradigm PipY. Life 12:1622. doi: 10.3390/life12101622, 36295057 PMC9605639

[ref85] Urwin L. Savva O. Corrigan R. M. (2024). Microbial primer: what is the stringent response and how does it allow bacteria to survive stress? Microbiology 170:483. doi: 10.1099/mic.0.001483, 39078282 PMC11288640

[ref86] Waldvogel A.-M. Pfenninger M. (2021). Temperature dependence of spontaneous mutation rates. Genome Res. 31, 1582–1589. doi: 10.1101/gr.275168.120, 34301628 PMC8415371

[ref87] Watanabe S. (2020). Cyanobacterial multi-copy chromosomes and their replication. Biosci. Biotechnol. Biochem. 84, 1309–1321. doi: 10.1080/09168451.2020.1736983, 32157949

[ref88] Watzer B. Spät P. Neumann N. Koch M. Sobotka R. Macek B. . (2019). The signal transduction protein PII controls ammonium, nitrate and urea uptake in Cyanobacteria. Front. Microbiol. 10:1428. doi: 10.3389/fmicb.2019.01428, 31293555 PMC6603209

[ref89] Weaver J. W. Proshkin S. Duan W. Epshtein V. Gowder M. Bharati B. K. . (2023). Control of transcription elongation and DNA repair by alarmone ppGpp. Nat. Struct. Mol. Biol. 30, 600–607. doi: 10.1038/s41594-023-00948-2, 36997761 PMC10191844

[ref90] Webster M. W. Chauvier A. Rahil H. Graziadei A. Charles K. Miropolskaya N. . (2024). Molecular basis of mRNA delivery to the bacterial ribosome. Science 386:eado8476. doi: 10.1126/science.ado8476, 39607923 PMC13040446

[ref91] Wells D. H. Long S. R. (2003). Mutations in *rpoBC* suppress the defects of a *Sinorhizobium meliloti relA* mutant. J. Bacteriol. 185, 5602–5610. doi: 10.1128/JB.185.18.5602-5610.2003, 12949113 PMC193748

[ref92] Zhang S.-R. Lin G.-M. Chen W.-L. Wang L. Zhang C.-C. (2013). ppGpp metabolism is involved in heterocyst development in the cyanobacterium *Anabaena* sp. strain PCC 7120. J. Bacteriol. 195, 4536–4544. doi: 10.1128/JB.00724-13, 23935047 PMC3807476

[ref93] Zuo Y. Wang Y. Steitz T. A. (2013). The mechanism of *E. coli* RNA polymerase regulation by ppGpp is suggested by the structure of their complex. Mol. Cell 50, 430–436. doi: 10.1016/j.molcel.2013.03.020, 23623685 PMC3677725

