# Supplementary figures and images for: Suppressor analysis in Synechococcus elongatus PCC7942 reveals key roles of (p)ppGpp in survival and nucleotide homeostasis
Source: Front Microbiol. 2026 Jun 3;17:1860886. doi: 10.3389/fmicb.2026.1860886 (PMC13273366; doi:10.3389/fmicb.2026.1860886)

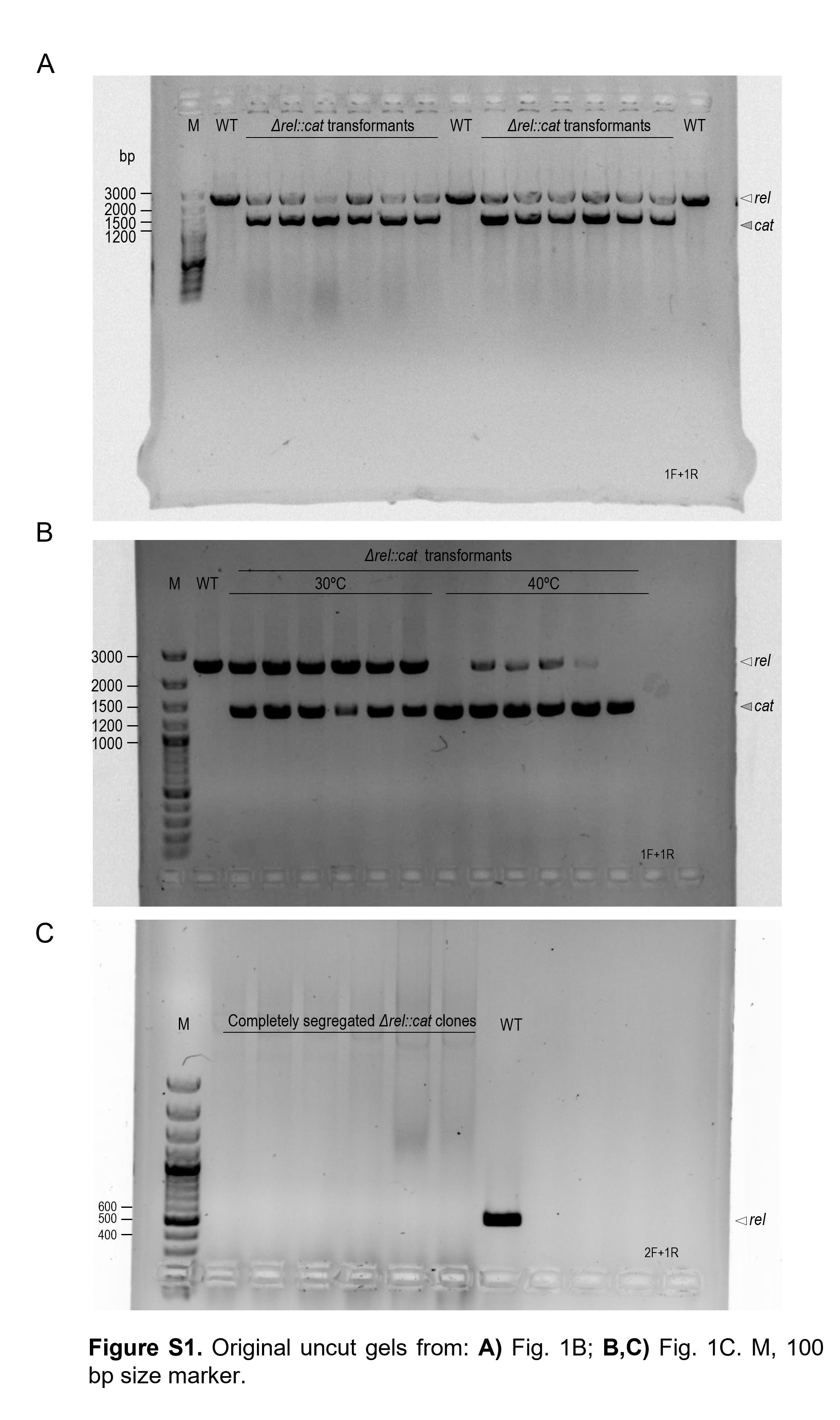

Supplement: Supplementary file 1 [file Image_1.tif]

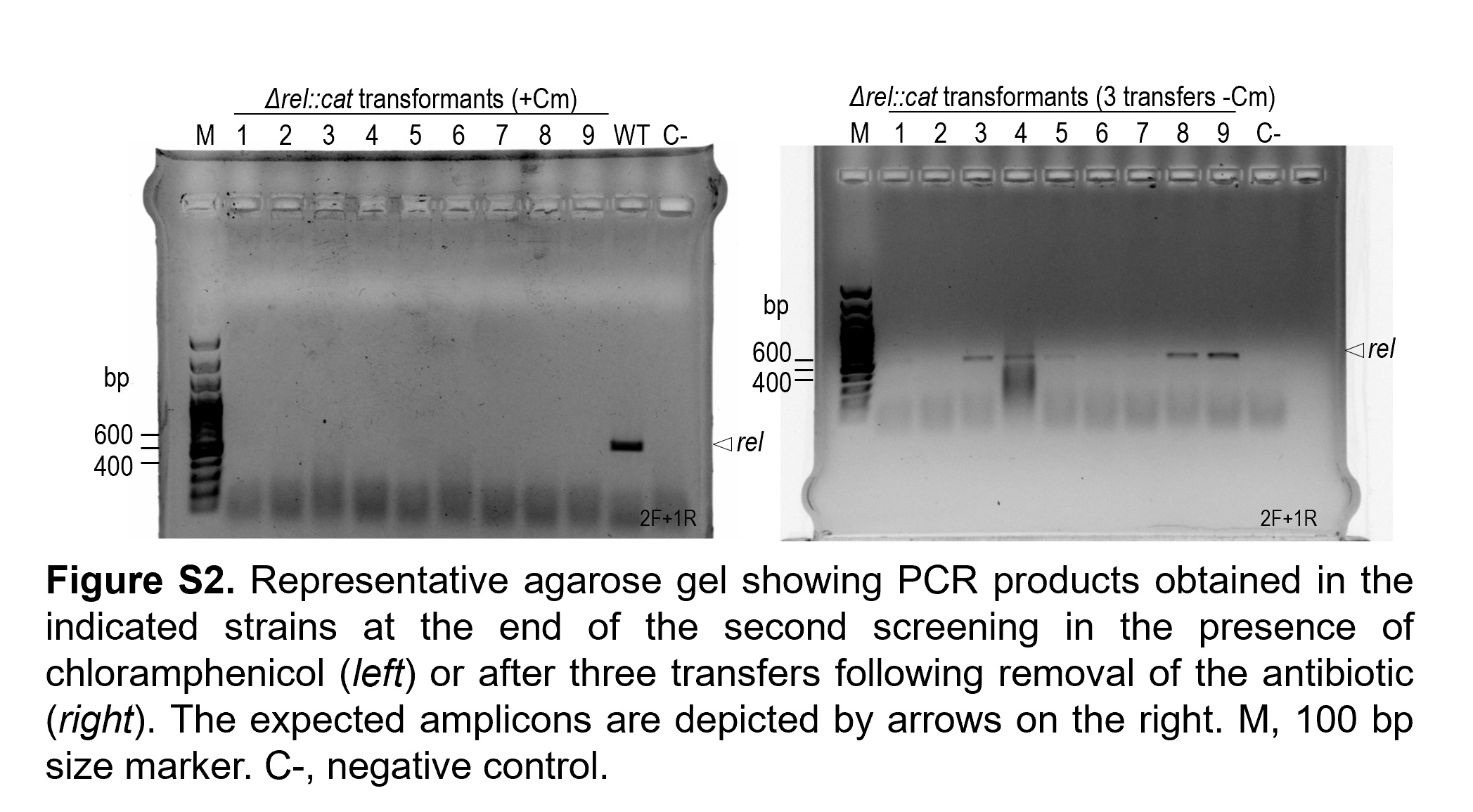

Supplement: Supplementary file 2 [file Image_2.tif]

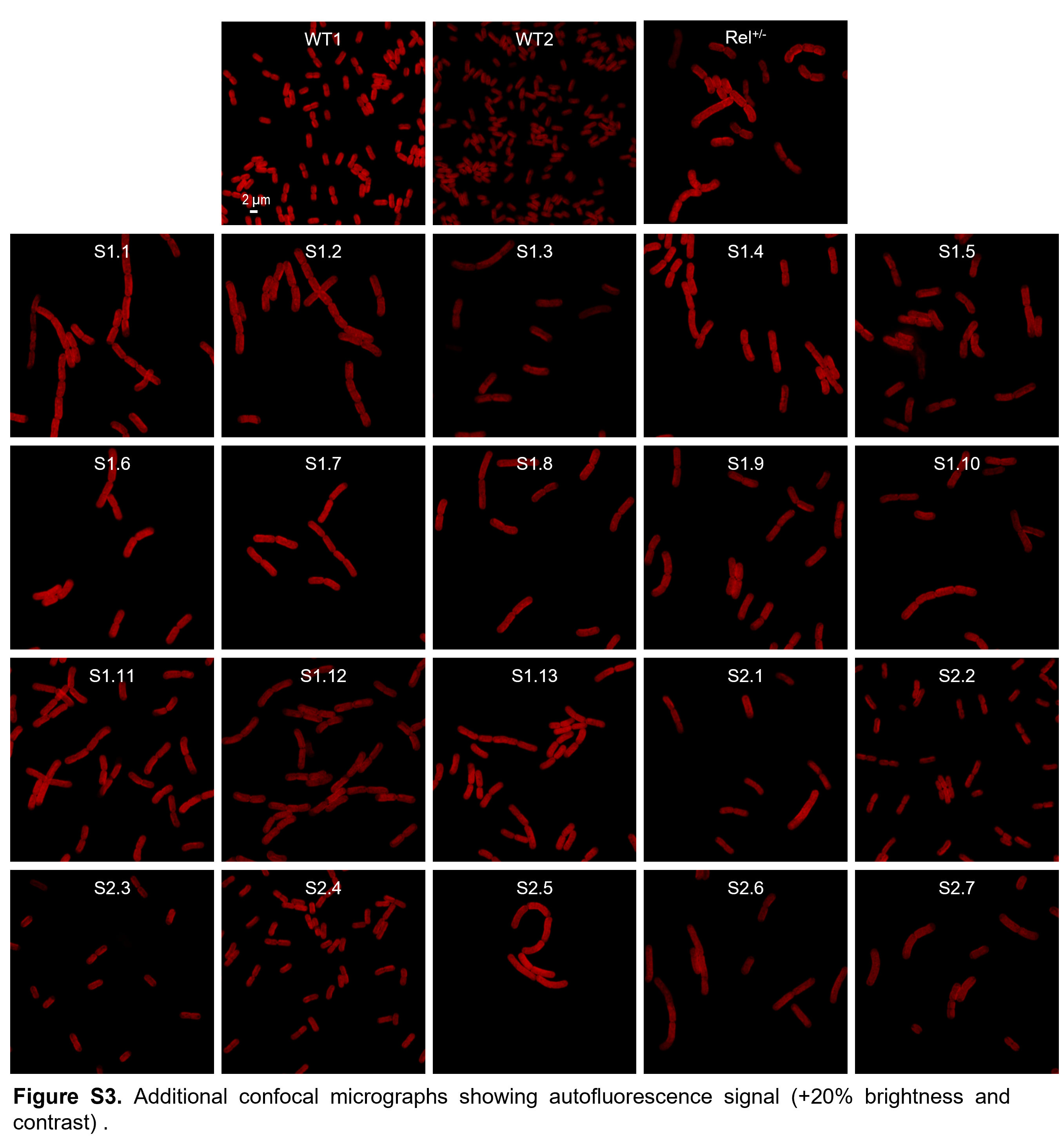

Supplement: Supplementary file 3 [file Image_3.tif]

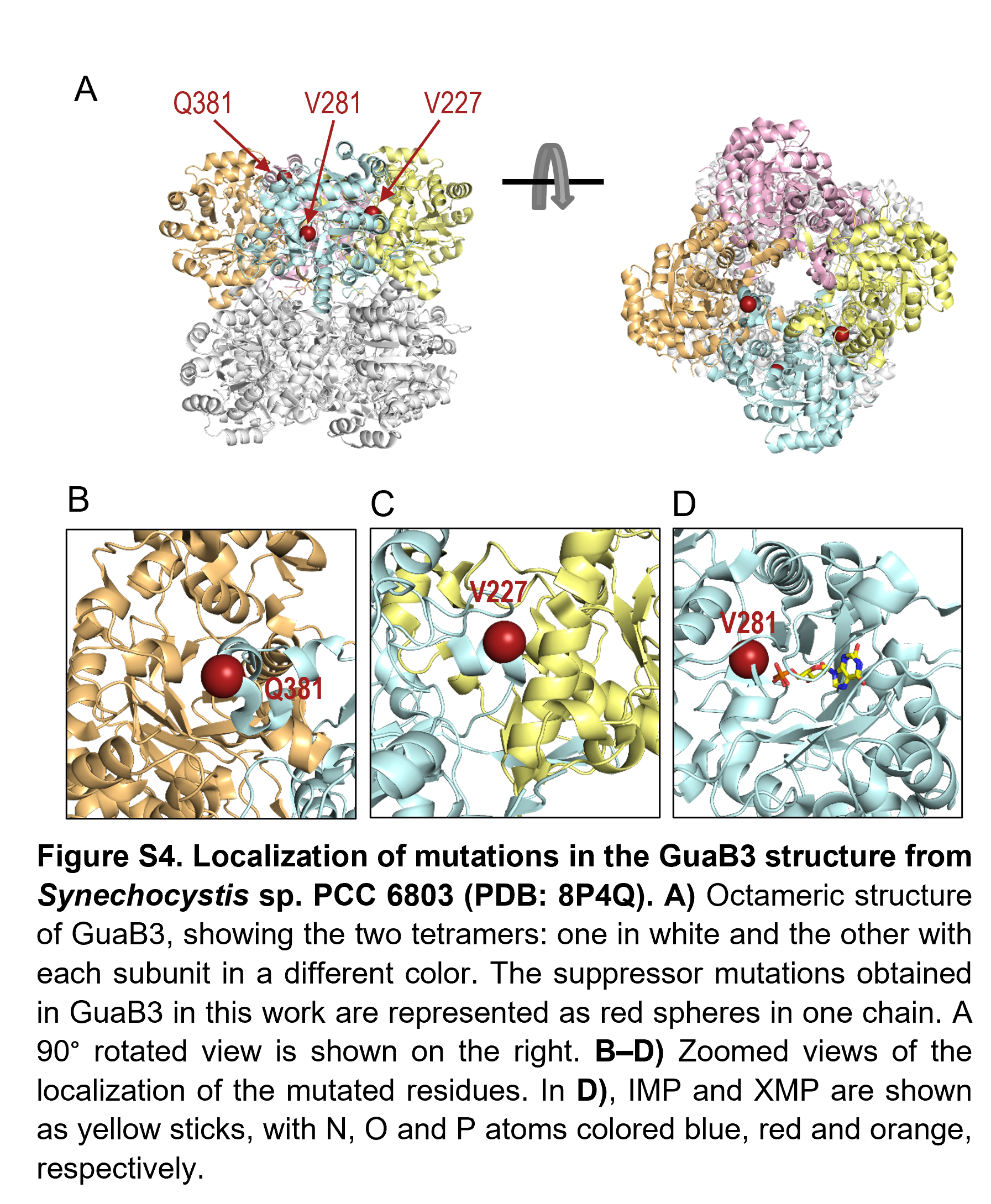

Supplement: Supplementary file 4 [file Image_4.tif]
