# Supplementary material for: Suppressor analysis in Synechococcus elongatus PCC7942 reveals key roles of (p)ppGpp in survival and nucleotide homeostasis
Source: Front Microbiol. 2026 Jun 3;17:1860886. doi: 10.3389/fmicb.2026.1860886 (PMC13273366; doi:10.3389/fmicb.2026.1860886)
Supplement: Supplementary file 5 [file Presentation_1.pptx]

## Slide 1
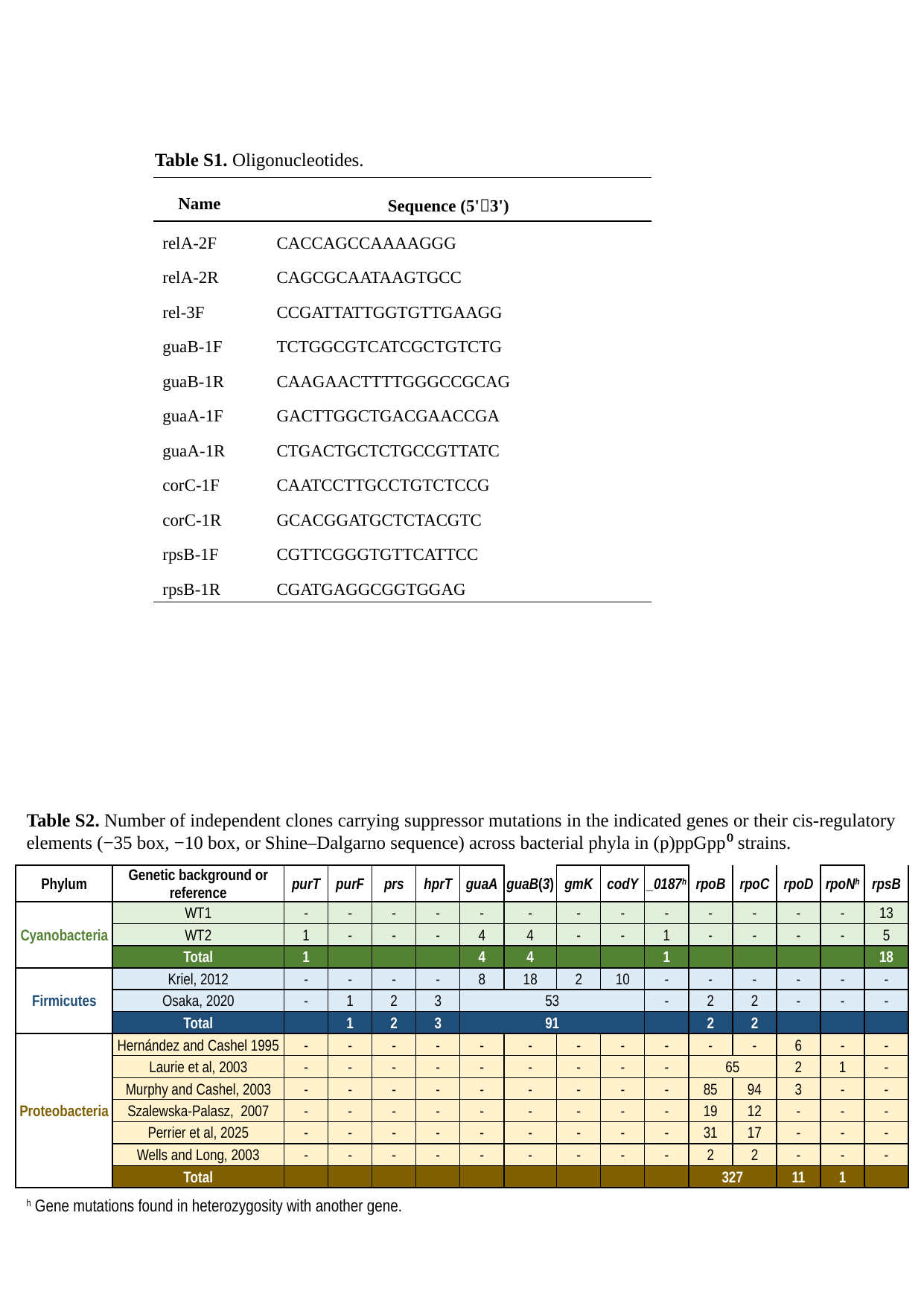

Table S1. Oligonucleotides.
| Name | Sequence (5'3') | |
| --- | --- | --- |
| relA-2F | | CACCAGCCAAAAGGG |
| relA-2R | | CAGCGCAATAAGTGCC |
| rel-3F | | CCGATTATTGGTGTTGAAGG |
| guaB-1F | | TCTGGCGTCATCGCTGTCTG |
| guaB-1R | | CAAGAACTTTTGGGCCGCAG |
| guaA-1F | | GACTTGGCTGACGAACCGA |
| guaA-1R | | CTGACTGCTCTGCCGTTATC |
| corC-1F | | CAATCCTTGCCTGTCTCCG |
| corC-1R | | GCACGGATGCTCTACGTC |
| rpsB-1F | | CGTTCGGGTGTTCATTCC |
| rpsB-1R | | CGATGAGGCGGTGGAG |
Table S2. Number of independent clones carrying suppressor mutations in the indicated genes or their cis-regulatory elements (−35 box, −10 box, or Shine–Dalgarno sequence) across bacterial phyla in (p)ppGpp⁰ strains.
| Phylum | Genetic background or reference | purT | purF | prs | hprT | guaA | guaB(3) | gmK | codY | \_0187h | rpoB | rpoC | rpoD | rpoNh | rpsB |
| --- | --- | --- | --- | --- | --- | --- | --- | --- | --- | --- | --- | --- | --- | --- | --- |
| Cyanobacteria | WT1 | - | - | - | - | - | - | - | - | - | - | - | - | - | 13 |
| | WT2 | 1 | - | - | - | 4 | 4 | - | - | 1 | - | - | - | - | 5 |
| | Total | 1 | | | | 4 | 4 | | | 1 | | | | | 18 |
| Firmicutes | Kriel, 2012 | - | - | - | - | 8 | 18 | 2 | 10 | - | - | - | - | - | - |
| | Osaka, 2020 | - | 1 | 2 | 3 | 53 | | | | - | 2 | 2 | - | - | - |
| | Total | | 1 | 2 | 3 | 91 | | | | | 2 | 2 | | | |
| Proteobacteria | Hernández and Cashel 1995 | - | - | - | - | - | - | - | - | - | - | - | 6 | - | - |
| | Laurie et al, 2003 | - | - | - | - | - | - | - | - | - | 65 | | 2 | 1 | - |
| | Murphy and Cashel, 2003 | - | - | - | - | - | - | - | - | - | 85 | 94 | 3 | - | - |
| | Szalewska-Palasz, 2007 | - | - | - | - | - | - | - | - | - | 19 | 12 | - | - | - |
| | Perrier et al, 2025 | - | - | - | - | - | - | - | - | - | 31 | 17 | - | - | - |
| | Wells and Long, 2003 | - | - | - | - | - | - | - | - | - | 2 | 2 | - | - | - |
| | Total | | | | | | | | | | 327 | | 11 | 1 | |
h Gene mutations found in heterozygosity with another gene.
